# Treatment Efficiency of Free and Nanoparticle-Loaded Mitoxantrone for Magnetic Drug Targeting in Multicellular Tumor Spheroids

**DOI:** 10.3390/molecules201018016

**Published:** 2015-09-30

**Authors:** Annkathrin Hornung, Marina Poettler, Ralf P. Friedrich, Jan Zaloga, Harald Unterweger, Stefan Lyer, Johannes Nowak, Stefan Odenbach, Christoph Alexiou, Christina Janko

**Affiliations:** 1 Department of Otorhinolaryngology, Head and Neck Surgery, Section for Experimental Oncology and Nanomedicine (SEON), Else Kröner-Fresenius-Stiftung Professorship, University Hospital Erlangen, Glückstraße 10a, 91054 Erlangen, Germany; E-Mails: annkathrin.hornung@uk-erlangen.de (A.H.); marina.poettler@uk-erlangen.de (M.P.); ralf.friedrich@uk-erlangen.de (R.P.F.); jan.zaloga@uk-erlangen.de (J.Z.); harald.unterweger@uk-erlangen.de (H.U.); stefan.lyer@uk-erlangen.de (S.L.); christoph.alexiou@uk-erlangen.de (C.A.); 2 Friedrich-Alexander-University Erlangen-Nuremberg (FAU), 91054 Erlangen, Germany; 3 Chair of Magnetofluiddynamics, Measuring and Automation Technology, Technische Universität Dresden, 01062 Dresden, Germany; E-Mails: johannes.nowak@tu-dresden.de (J.N.); stefan.odenbach@tu-dresden.de (S.O.)

**Keywords:** nanomedicine, magnetic drug targeting, superparamagnetic iron oxide nanoparticles, multicellular tumor spheroids, chemotherapy

## Abstract

Major problems of cancer treatment using systemic chemotherapy are severe side effects. Magnetic drug targeting (MDT) employing superparamagnetic iron oxide nanoparticles (SPION) loaded with chemotherapeutic agents may overcome this dilemma by increasing drug accumulation in the tumor and reducing toxic side effects in the healthy tissue. For translation of nanomedicine from bench to bedside, nanoparticle-mediated effects have to be studied carefully. In this study, we compare the effect of SPION, unloaded or loaded with the cytotoxic drug mitoxantrone (MTO) with the effect of free MTO, on the viability and proliferation of HT-29 cells within three-dimensional multicellular tumor spheroids. Fluorescence microscopy and flow cytometry showed that both free MTO, as well as SPION-loaded MTO (SPION^MTO^) are able to penetrate into tumor spheroids and thereby kill tumor cells, whereas unloaded SPION did not affect cellular viability. Since SPION^MTO^ has herewith proven its effectivity also in complex multicellular tumor structures with its surrounding microenvironment, we conclude that it is a promising candidate for further use in magnetic drug targeting *in vivo*.

## 1. Introduction

Nanomedicine offers fascinating opportunities for new medical treatments and diagnosis. Here, especially superparamagnetic iron oxide nanoparticles (SPION) have received scientific interest in tissue engineering, drug delivery, hyperthermia and magnetic resonance imaging techniques [1,2]. In addition, these particles can be used as a platform for targeted delivery of drugs to the desired region, guided by an external magnetic field, referred to as “magnetic drug targeting” (MDT) [3,4,5,6]. MDT has been used for the accumulation of chemotherapeutics, antibiotics or thrombolytic agents in tumors, infection sites or thrombosis [7,8,9], where the specific accumulation of drugs in the desired region increases therapeutic efficacy and reduces adverse side effects compared to systemic application [10]. Especially for cancer therapy, in the last few years, numerous promising SPION systems with various drug loadings have been developed for the translation from bench to bedside [11,12]. Therapeutic success using MDT *in vivo* has already been shown in the treatment of tumor-bearing rabbits employing mitoxantrone (MTO)-loaded iron oxide nanoparticles [13,14].

Although this new technology can offer great therapeutic advantages, the increasing applicability of drug-loaded nanoparticles requires detailed knowledge of their therapeutic and toxicological impact. So far, numerous studies, showing the effective killing of tumor cells by chemotherapeutics-loaded nanoparticles, exist [15,16]. However, most of the current knowledge on the effects of nanoparticles on cellular physiology is derived mainly from monolayer cell culture studies, which might have limited ability to reveal the interaction of those particles with complex physiological tissues. In the last few years, many new drugs have been withdrawn during animal trials since *in vitro* toxicity analysis failed to identify their hazards [17]. To receive data from model systems with higher physiological relevance, there has been increasing emphasis on three-dimensional (3D) cell cultures, since cells growing in spheroids show a higher degree of morphological and functional differentiation [18]. Additionally, it has become clear that resistance to radiotherapy and chemotherapy in cancer treatment might result from micro-environmental factors [19,20]. Thus, multicellular spheroids mimicking the tumor environment might be more suitable for the assessment of efficacy of therapeutic agents than monolayer cell cultures [21], which only poorly predict a drug’s therapeutic outcome *in vivo* [22]. Especially for nanotoxicological investigations, the mass transfer gradient within the different cellular layers of a spheroid might play an important role [23]. Recently, it has been shown that the inflammatory potential and cytotoxicity of ZnO nanoparticles affect the outer layer of a spheroid more dramatically, whereas the inner cell layers are protected [24]. Importantly, the toxic dose ranges for some drugs obtained in 3D cell cultures were very similar to those from animal experiments [25,26,27], demonstrating that this advanced cell culture system might be able to bridge the gap between 2D cell culture and *in vivo* testing [28,29].

The aim of this study was to analyze the effectivity of in-house fabricated MTO-loaded SPION for MDT in multicellular tumor spheroids as a model system for small solid tumors. A standardized multicellular 3D tumor model using HT-29 colon carcinoma cells was established previously [30]. Based on the first microscopic observations of spheroid morphology after MTO treatment, more advanced readouts for viability, apoptosis and necrosis were evaluated for cells growing in tumor spheroids in this study. A further goal was to achieve information about penetration and effect of free MTO, nanoparticle-loaded MTO and unloaded nanoparticles in 3D multicellular structures.

## 2. Results and Discussion

### 2.1. Spheroid Growth and Cellular Proliferation of Untreated Tumor Spheroids

**Figure 1:**
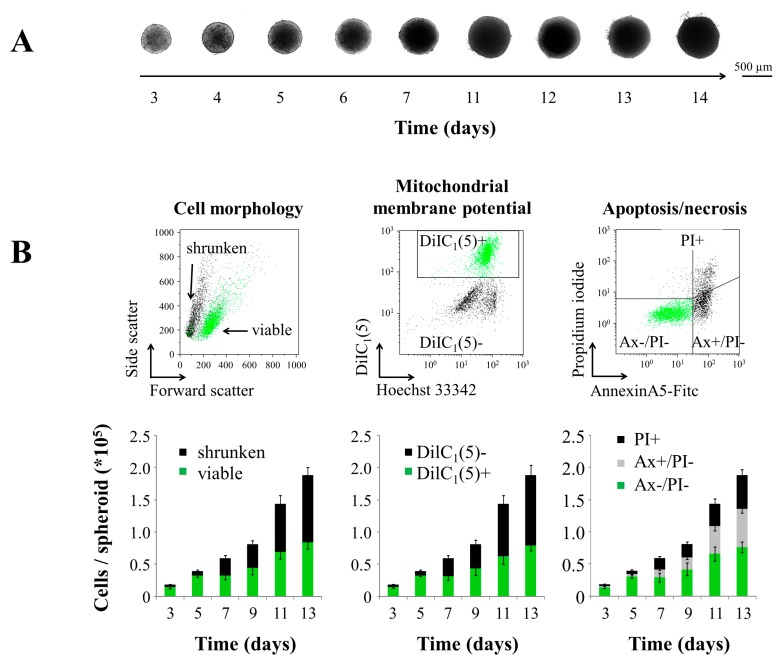

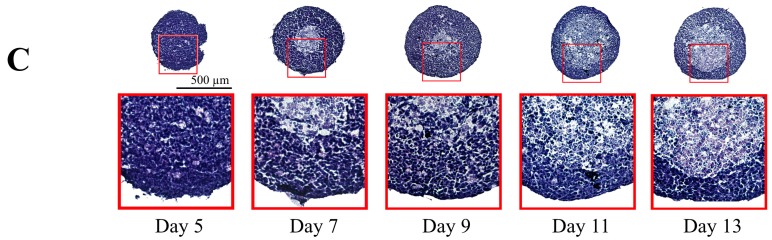
Growth and cellular proliferation of HT-29 tumor spheroids. (**A**) Transmission microscopy of representative spheroids; (**B**) flow cytometry of single-cell suspensions prepared from tumor spheroids; raw data files exemplarily show the cells 11 days after seeding. Left: morphological cell analysis by forward/side scatter (FSC/SSC) reflects cellular size and granularity. Middle: analysis of mitochondrial membrane potential using the cyanine dye DiIC_1_(5); DiIC_1_(5)+ cells are considered viable; DiIC_1_(5)− cells are dying/dead. Right: Annexin A5-FITC (Ax) and propidium iodide staining (PI) discriminated between viable (Ax−/PI−), apoptotic (Ax+/PI−) and necrotic (Ax+/PI+) cells. Shown are the mean values of cells from *n* = 10 spheroids with standard deviations; (**C**) Hematoxylin/eosin stainings of cryosections; magnifications show the proliferative layer and necrotic cores of the spheroids.

Based on previous experiments establishing optimal culture conditions for the formation of HT-29 spheroids, 6000 cells were seeded into agarose-coated 96-well plates. Investigations started 72 h after seeding, when cells had already formed dense spheroid structures and showed consistent and reproducible growth. Transmission microscopy revealed that the spheroids initially grew very fast, but reduced growth after prolonged incubation (Figure 1A).

For more detailed information about cell viability within tumor spheroids, flow cytometry was performed of single-cell suspensions prepared from those structures (Figure 1B). Cells were stained for mitochondrial membrane potential using 1,1′,3,3,3′,3′-hexamethylindodicarbo-cyanine iodide (DiIC_1_(5)), phosphatidylserine exposure using Annexin A5-FITC (Ax) and plasma membrane integrity using propidium iodide (PI) [31,32]. The membrane-permeable DNA dye Hoechst 33342 discriminates between DNA-containing cells (Hoechst positive) and debris/nanoparticles, having no Hoechst fluorescence. Forward scatter and side scatter delivered information about cellular size and granularity. In combination, those markers provided a comprehensive picture of cell viability and death. Figure 1B summarizes the data from *n* = 10 spheroids, whereas viable cells are depicted in green, and dying/dead cells are depicted in grey/black. Data from all three readouts are concordant, showing that with prolonged incubation time, the total amount of cells within the spheroids increases due to cellular proliferation. During the observation period, however, a remarkable amount of cells shows a dying/dead phenotype reflected by an altered cell morphology (shrunken), loss of mitochondrial membrane potential (DiIC_1_(5)−), exposure of phosphatidylserine (Ax+) and/or loss of plasma membrane integrity (PI+).

To localize viable and dead cells within the spheroids, cryosections of the spheroids were prepared. Hematoxylin/eosin (HE) staining clearly indicated that the morphology of the cells in the center changed over time (Figure 1C). After three days of incubation, the HT-29 spheroids appeared as a homogenous cell package, whereas with prolonged incubation, a necrotic core of increasing size developed, which might be due to limitations in nutrient and oxygen transport and accumulation of metabolic waste. Thus, larger spheroids contain a proliferating cell layer on the surface, an area of viable quiescent cells underneath and a necrotic core in the center [33]. Highly proliferating cells are the target of most cancer therapies, and thus, many anti-neoplastic drugs have only limited toxicity against slowly/non-proliferating cells, as they occur in the center of a tumor [34,35]. Notably, hypoxic areas within cancers have been identified as one cause of drug resistance *in vivo* [36]. As an intermediary model between monolayer cell cultures and complex tumors *in vivo*, spheroids seem to be suitable for advanced *in vitro* investigations of nanoparticles for tumor treatment.

### 2.2. Penetration of MTO into 3D Multicellular Tumor Spheroids

Since penetration of the drug in tumor tissues may be significantly altered by the properties of the delivery vehicle [37,38], we compared the ability of free MTO and nanoparticle-bound MTO to penetrate into 3D cellular structures. As a nanoparticle system, in-house fabricated SPIONs with a lauric acid (LA) layer and a stabilizing protein corona of bovine serum albumin (BSA) were used, which are called SEON^LA-BSA^ (Section of Experimental Oncology and Nanomedicine (SEON)), and after loading with the cytotoxic drug MTO, they are named SEON^LA-BSA*MTO^, respectively. These particles were extensively physico-chemically characterized previously (Table 1) [12,39].

**Table 1:** Summary of physico-chemical properties of unloaded superparamagnetic iron oxide nanoparticles (SEON^LA-BSA^) and mitoxantrone loaded ones (SEON^LA-BSA*MTO^) in RPMI 1640 cell culture medium containing 10% FCS. SEON, Section of Experimental Oncology and Nanomedicine; LA, lauric acid; BSA, bovine serum albumin; MTO, mitoxantrone.

	SEON^LA-BSA^	SEON^LA-BSA*MTO^
Core diameter (TEM) (nm) in water	7.64 ± 1.68	
Hydrodynamic diameter (DLS) (nm)	61.7 ± 1.15	72.7 ± 0.23
Zeta potential (mV)	−12.9 ± 0.55	−10.17 ± 0.80
Polydispersity index	0.346 ± 0.028	0.298 ± 0.002

**Figure 2:**
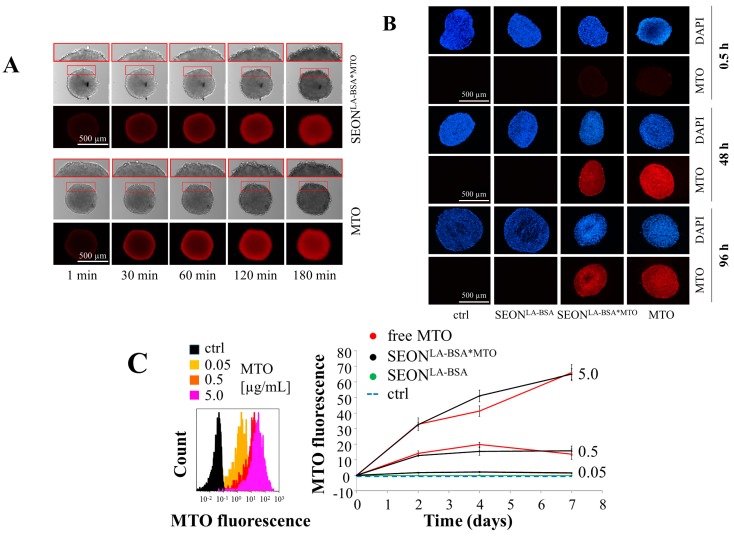
Penetration of free mitoxantrone (MTO), SEON^LA-BSA*MTO^ and unloaded SEON^LA-BSA^ into tumor spheroids. Due to its inherent fluorescence (excitation 638 nm, emission 725 nm), MTO penetration can be observed by fluorescence microscopy and flow cytometry. (**A**) Live cell imaging of spheroids treated with MTO. Spheroids were monitored for several hours; transmission and MTO fluorescence pictures were extracted at the indicated time points. MTO concentration of free MTO and SEON^LA-BSA*MTO^ each: 5.0 µg/mL; (**B**) Cryosections of MTO-treated spheroids were stained with DAPI. MTO concentration of free MTO and SEON^LA-BSA*MTO^ each: 0.5 µg/mL; (**C**) Flow cytometry of single-cell suspensions prepared from tumor spheroids; left: raw data of MTO fluorescence for spheroids three days after treatment; right: dose- and time-dependent kinetics of MTO uptake. Equal concentrations of free MTO and nanoparticle-bound MTO were tested; the MTO concentrations are given (5.0, 0.5 and 0.05 µg/mL); as controls, unloaded nanoparticles in iron concentrations corresponding to the drug-loaded nanoparticles were tested. Shown are the mean values of *n* = 6 spheroids with standard deviations.

Due to the inherent fluorescence of MTO, the analysis of cellular uptake is easily feasible via flow cytometry and fluorescence microscopy [10,40]. Spheroids were incubated with free MTO or nanoparticle-loaded MTO (SEON^LA-BSA*MTO^), as well as unloaded nanoparticles (SEON^LA-BSA^) with iron concentrations corresponding to that in MTO-loaded nanoparticles. Mock-treated cells served as controls. Live cell imaging showed that free MTO, as well as nanoparticle-bound MTO have the ability to quickly penetrate into the outer layers of the spheroid (Figure 2A). In both cases, a clear time-dependent enrichment of the chemotherapeutic agent in the spheroid was observed. Notably, these high MTO concentrations (5 µg/mL) induce a fast alteration of the cell morphology at the outer spheroid layers, as shown in transmission microscopy. To analyze the penetration into deeper layers and the center, cryosections of drug-incubated spheroids were prepared, stained with the nuclear dye DAPI and analyzed in fluorescence microscopy for MTO and DAPI fluorescence (Figure 2B). Sections of spheroids 0.5 h, 48 h and 96 h after treatment reveal a time-dependent penetration of MTO into the spheroids, even in deeper cellular layers. Flow cytometry confirmed the time and dose-dependent penetration of free, as well as nanoparticle-loaded MTO into cells within spheroids, whereas untreated cells and cells treated with unloaded nanoparticles displayed no MTO fluorescence (Figure 2C). It has been shown previously that various nanoparticles might have limited accumulation in the center of the tumor due to inefficient spheroid penetration [41,42]. However, from our data we conclude that all cells in the spheroid have the possibility to get in contact with the chemotherapeutic agent. Due to their more physiological cell-cell contact geometry, mechanical properties, extracellular matrix and mass transport, multicellular tumor spheroids can serve as a valuable model in drug penetration studies [43].

### 2.3. Impact of Free MTO, SEON^LA-BSA^ and SEON^LA-BSA*MTO^ on Cell Viability

**Figure 3:**
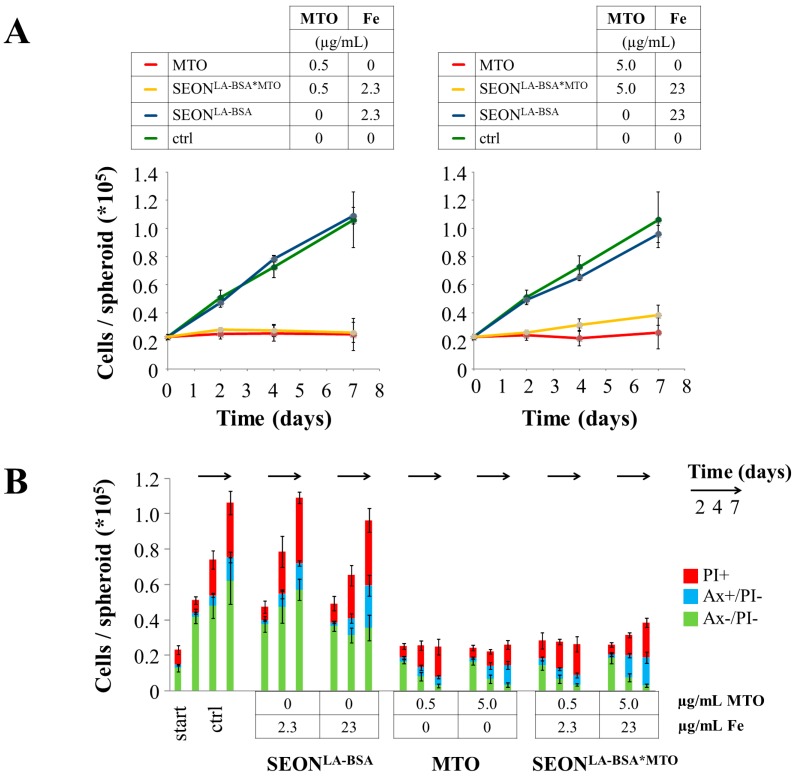

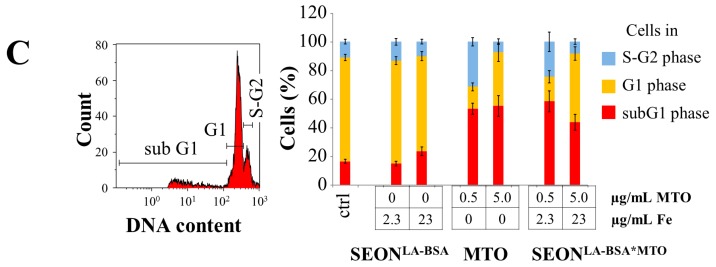
Cell viability of HT-29 tumor spheroid cells after treatment with free MTO, SEON^LA-BSA*MTO^ and unloaded SEON^LA-BSA^. Equal concentrations of free MTO and nanoparticle-bound MTO were tested; as controls, unloaded nanoparticles in the concentrations corresponding to the drug-loaded nanoparticles were used. Proliferation/viability of cells within the spheroids was analyzed from single-cell suspensions using MUSE Cell Analyzer (**A**) or flow cytometry (**B**,**C**); (**B**) Annexin A5-FITC (Ax) and propidium iodide staining (PI) discriminate between viable (Ax−/PI−), apoptotic (Ax+/PI−) and necrotic (PI+) cells; (**C**) PI-Triton staining provides information about cell cycle and DNA degradation. Left: raw data of control cells at *t* = 4 days; right: cells at *t* = 7 days. S-G2: cells with replicating DNA; G1: cells with diploid DNA; subG1: cells with degraded DNA. Shown are the mean values of cells from *n* = 6 spheroids with standard deviations.

As shown, both free MTO and SEON^LA-BSA*MTO^ are capable of penetration into HT-29 tumor spheroids. Previous observations in transmission microscopy revealed that the growth of the spheroids was dose dependently inhibited by MTO treatment [30]. In concordance with these early observations, analysis of cell counts indicated a complete inhibition of proliferation in the presence of MTO (Figure 3A), whereas unloaded nanoparticles did not inhibit cellular proliferation compared to untreated control cells. Ax/PI stainings of single-cell suspensions prepared from tumor spheroids indicated that not only cell proliferation was inhibited, but also cell death was dose- and time-dependently induced by free MTO and SEON^LA-BSA*MTO^ (Figure 3B). During the observation period of seven days, first apoptosis and later secondary necrosis were induced by the drug. Analysis of the DNA using PI-Triton staining showed a block in the cell cycle in the G2-phase, as well as degradation of the DNA after incubation with free MTO and SEON^LA-BSA*MTO^ for seven days (Figure 3C). Altogether, free MTO and SEON^LA-BSA*MTO^ showed comparable efficacies in the same doses. Previous experiments, investigating the effect of free MTO and SEON^LA-BSA*MTO^ in suspension cells and monolayer cell culture, were in line with these results [12,15], indicating that no cytotoxic activity of MTO is lost by loading onto SEON^LA-BSA^. Therefore, SEON^LA-BSA*MTO^ might be a valuable nanoparticle system for future use in cancer therapy.

When comparing the penetration of free MTO and nanoparticle-loaded MTO into spheroids, a clear dose-dependent effect is found (Figure 2C). However, despite the different total amount of MTO within the cells of the spheroid, no concentration-dependent effect in the inhibition of cell proliferation (Figure 3A) or cell death induction (Figure 3B) has been shown. We speculate that even the use of lower MTO concentrations leads to an excess of MTO, especially on the cells of the small proliferating outer layer of the spheroid. Comparing the effectivity of drug-loaded nanoparticles in monolayer cell cultures and in spheroids, it has been shown previously that significantly lower concentrations of drugs induce toxic effects in cell monolayers compared to three-dimensional structures [42,44,45], probably because monolayer cultures mainly consisting of proliferating cells are more susceptible to chemotherapeutic drugs. Furthermore, the extracellular matrix and differences in cell adhesion might protect the spheroids from further damage caused by the chemotherapeutic agent. It has been shown previously that the 3D matrix adhesion differs from 2D cell culture adhesion in structure and function [46]. Altogether, these diverging results of drug penetration/efficacy of chemotherapeutic-loaded nanoparticles in 2D and 3D cell culture systems emphasize the relevance of 3D cell cultures, especially in nanotoxicological research.

## 3. Experimental Section

### 3.1. Synthesis of Unloaded and Chemotherapeutics Loaded Superparamagnetic Iron Oxide Nanoparticles

SPIONs were synthesized at the Section of Experimental Oncology and Nanomedicine (SEON), University Hospital Erlangen, as described previously [12]. For stabilization, they were coated with lauric acid (LA) and covered by a protein corona of bovine serum albumin (BSA). The resulting SEON^LA-BSA^ were then loaded with the chemotherapeutic drug mitoxantrone (MTO, TEVA Pharma, Ulm, Germany), resulting in SEON^LA-BSA*MTO^. SEON^LA-BSA^ and SEON^LA-BSA*MTO^ are stable in cell culture medium and blood. When SEON^LA-BSA*MTO^ are incubated in cell culture medium, MTO is very slowly released from the particles; after 72 h, more than 90% of the original amount of MTO is still bound to the particles. SEON^LA-BSA^ and SEON^LA-BSA*MTO^ were extensively physico-chemically characterized previously [12,39]; Table 1 provides a summary of the basic physico-chemical nanoparticle characteristics.

### 3.2. Cells, Culture Conditions and Generation of Spheroids

The colon carcinoma cell line HT-29 (ATCC/LGC GmbH, Wesel, Germany) was cultured in McCoy’s 5A medium (Gibco^®^, Life Technologies GmbH, Darmstadt, Germany) supplemented with 10% fetal calf serum (FCS) under standard cell culture conditions in a humidified incubator (INCOmed, Schwabach, Memmert, Germany) at 37 °C and 5% CO_2_. For the experiments, the cells were grown to a confluence of 80%–90% (approximately 15 × 10^6^ cells in flasks with 75 cm^2^ surface area) and passaged twice a week using 0.25% trypsin/0.02% EDTA in PBS (PAN Biotech, Aidenbach, Germany).

For spheroid generation, each well of a 96-well plate (Sarstedt, Nümbrecht, Germany) was pre-coated with 30 µL 1.5% agarose gel (Roth, Karlsruhe, Germany). The cells were detached with 2 mL 0.25% trypsin/0.02% EDTA in PBS from the cell culture flasks and resuspended in 10 mL medium to form single-cell suspensions. Then, cells were counted, and viability was determined using MUSE^®^ Cell Analyzer (Merck-Millipore, Billerica, MA, USA). Six thousand cells in 200 µL medium were seeded into each agarose coated well; for optimal cell distribution, the plates were rotated several times. Finally, the plates were incubated for >72 h to form tightly-packed multicellular tumor spheroids.

### 3.3. Transmission Microscopy

Growth of the spheroids was investigated using an Axiovert 40 CFL Microscope (Zeiss, Jena, Germany) with a 2.5× objective. Regularly, pictures of the spheroids were taken with the Axio Vision SE64 Rel4.9 software (Zeiss). The size of the spheroids was assessed using ImageJ software (National Institutes of Health, Bethesda, MD, USA) and an objective calibration slide in order to calculate the spheroid area. Data analysis was performed in MS Excel.

### 3.4. Cryosections of HT-29 Spheroids

For each treatment condition, 38 HT-29 spheroids were harvested from the 96-well plate with a cropped pipette and all collected in a 15-mL Falcon (Sarstedt). Spheroids were centrifuged at 300 *g* for 3 min, and the supernatant was removed. Spheroids were embedded in SLEE CRYOGLUE Embedding medium (SLEE medical GmbH, Mainz, Germany) and frozen for at least 20 min at −80 °C. Spheroid sections with a thickness of 10 µm were prepared using an MNT microtome (SLEE medical GmbH) and placed on a Thermo scientific glass object slide (Menzel GmbH, Braunschweig, Germany).

### 3.5. Hematoxylin and Eosin Staining of Spheroid Sections

The sections were washed with distilled water and stained with Hematoxylin Gill III (Merck KGaA, Darmstadt, Germany) for 6 min. Then slides were treated with 0.1% HCl (Carl Roth GmbH+Co.KG, Karlsruhe, Germany) and placed under running tap water for 3 min. Afterwards, sections were counterstained with 0.5% eosin (Carl Roth GmbH + Co.KG) for 6 min. Slides were briefly rinsed with tap water and then dehydrated in an ascending alcohol solution of 70% ethanol two times for 2 s, 96% ethanol two times for 2 min and xylol two times for 3 min. The sections were mounted in DPX mounting medium (Merck KGaA) and analyzed with an Axio Observer Z.1 microscope (Zeiss)

### 3.6. Analysis of MTO Penetration into the Spheroids by Fluorescence Microscopy of Cryosections

To investigate the drug penetration of MTO and SEON^LA-BSA*MTO^ into HT-29 spheroids, spheroids were frozen at distinct time points, and cryosections were prepared as described previously. The sections were mounted with DAPI containing mounting media (Sigma-Aldrich, St Louis, MO, USA), and MTO and DAPI fluorescence was assessed by an Axio Observer Z.1 microscope (Zeiss).

### 3.7. Analysis of MTO Penetration into the Spheroids by Live Cell Imaging

For live cell imaging, two spheroids were harvested from the agarose coated wells and transferred into chamber slides (Thermo Fisher Scientific, Rochester, NY, USA). Then, 400 µL medium were added, and spheroids were treated with MTO and the corresponding amount of SEON^LA-BSA*MTO^. Over a period of 3 h every 3 min, images of the spheroids were taken by an Axio Observer Z.1 microscope (Zeiss).

### 3.8. Harvesting of Spheroids and Preparation of Single-Cell Suspensions

For each experimental condition, 8 spheroids were harvested from the agarose coated wells and transferred to empty 96-well plates. Spheroids were incubated in 100 µL trypsin for 10–20 min at room temperature. To support dissolving of the spheroids, every 5 min, cells were pipetted up and down. The dissociation of the spheroids into single cells was monitored by transmission microscopy. By adding FCS-containing medium, the process was stopped, and the cell suspension and the supernatant of the well were transferred into 1.5 mL Eppendorf tubes and centrifuged with 300 *g* for 5 min. The supernatant was removed, and cells were resuspended in 90 µL medium.

### 3.9. Analysis of Viability and Drug Penetration into Cells in Flow Cytometry

Forty-microliter aliquots of the single-cell suspensions of the spheroids were incubated with 60 µL MUSE staining mix and counted with the MUSE^®^ Cell Analyzer (EMD Millipore Corporation, Hayward, CA, USA). Absolute numbers of total and viable cells were achieved by this measurement.

In parallel to the MUSE measurements, 50-µL aliquots of the single-cell suspensions were stained with 250 µL freshly-prepared staining mix consisting of 1 µg/mL AnnexinA5-FITC (Ax-FITC), 1 µg/mL Hoechst 33342 (both from Life Technologies), 2 µg/mL propidium iodide (PI, Sigma-Aldrich, Taufkirchen, Germany) and, for some measurements, 0.4 µL DiIC_1_(5) per 1 mL Ringer’s Solution (Baxter Healthcare SA, Zurich, Switzerland). Cells were incubated in the mixture for 20 min at 4 °C. The fluorescences were measured with a Gallios flow cytometer (Beckman Coulter, Fullerton, CA, USA). Excitation for both FITC and PI was at 488 nm; the FITC fluorescence was recorded on the FL1 sensor (525/38 nm band pass filter, BP); the PI fluorescence on the FL3 sensor (620/30 nm BP); the DiIC_1_(5) fluorescence was excited at 638 nm and recorded on the FL6 sensor (675/20 BP); and the Hoechst fluorescence was excited at 405 nm and recorded on the FL9 sensor (430/40 nm BP). The MTO fluorescence was excited at 638 nm and recorded by the FL7 sensor (725/20 nm BP). Electronic compensation was used to eliminate fluorescence bleed through. Data analysis was performed with Kaluza software Version 1.2 (Beckman Coulter).

### 3.10. Analysis of Cell Cycle and DNA Degradation in Flow Cytometry

Single-cell suspensions of HT-29 spheroids were prepared as described above. The cell pellet was resuspended in 500 µL medium. Cells were fixed by adding 3 mL of 70% (*v*/*v*) ice-cold ethanol (Carl Roth GmbH + Co.KG) stored at −20 °C for further processing.

The cell suspension was then centrifuged (400 g for 5 min); the supernatant was removed, and the cells were washed in 5 mL PBS and centrifuged again. Then, 0.5 mL of PBS and 0.5 mL DNA extraction buffer (192 mL of 0.2 M Na_2_HPO_4_, 8 mL 0.1% Triton X-100 (*v*/*v*), pH 7.8) were added and incubated for 5 min at room temperature. Cells were centrifuged; the supernatant was removed, and cells were resuspended in 500 µL of DNA staining solution (1 mg/mL sodium citrate, 0.1% Triton X-100 (*v*/*v*) and 50 μg/mL PI in water) and incubated at room temperature for 30 min in the dark. The PI fluorescence was analyzed in flow cytometry [47].

## 4. Conclusions

In summary, by analyzing the amount of viable, apoptotic and necrotic cells, we were able to quantify the effect of SEON^LA-BSA*MTO^ in comparison to free MTO in small solid tumor spheroids. It became clear that binding of the chemotherapeutic drug to this vehicle system does not influence the therapeutic efficacy. Moreover, the high anti-proliferative and anti-tumorigenic potential of SEON^LA-BSA*MTO^, which has been previously demonstrated in 2D cell culture experiments, was confirmed in 3D cell culture. On the other hand, unloaded SEON^LA-BSA^ showed a high biocompatibility and did not influence the growth of the spheroids. By applying 3D cell culture, further information about the penetration and uptake of the chemotherapeutic drug has been achieved. With fluorescence microscopy, we were able to track the path of MTO and SEON^LA-BSA*MTO^ into the inner center of the tumor spheroid over a period of time.

Altogether, since tumors are three-dimensional structures with inhomogeneous cellular phenotypes also dependent on their location within the tumor, 3D cell culture spheroids may represent a bridge between 2D cell cultures and tumors. 2D cell systems already offer a huge range of cell-based assays for drug analysis, as they have become the basic step in the process of drug investigation. However, they are a highly artificial tool and have only limited explanatory power. Here, the 3D tumor spheroid system offers perspectives and might be a valuable tool for standardized monitoring of local drug penetration capacity, binding properties and tissue distribution of therapeutic drugs in a pathophysiological and multicellular context. At the same time, it can help to minimize animal experiments according to the 3R principles: “reduce”, “replace” and “refine”. In order to implement 3D culture systems into routine workflows of nanoparticle and drug approval, the establishment and validation of an easy to handle, predictable analytic tool is a clear goal for the future.

Finally, our study revealed that 3D cell culture systems offer a highly promising instrument for *in vitro* nanotoxicology testing and the implementation of MDT into clinical practice. The influence of a magnetic field on nanoparticle targeting and cell death kinetics will be part of future investigations.
